# Primaquine-derived 5,6-orthoquinone concentrations in urine associated with CYP2D6 genotype-predicted phenotype and blood methemoglobin levels

**DOI:** 10.1128/aac.00539-26

**Published:** 2026-07-13

**Authors:** Mario Vernandes, Willy Agustine, Ihsan Fadilah, Michael Christian, Angela Rumaseb, Decy Subekti, Melva Louisa, Ric N. Price, Kamala Thriemer, J. Kevin Baird

**Affiliations:** 1 Faculty of Medicine, Oxford University Clinical Research Unit Indonesia, Universitas Indonesia 95338, Jakarta, Indonesia; 2 Centre for Tropical Medicine and Global Health, Nuffield Department of Medicine, University of Oxford 270459, Oxford, United Kingdom; 3 Division of Global and Tropical Health, Menzies School of Health Research and Charles Darwin University 266967, Darwin, Australia; 4 Department of Pharmacology and Therapeutics, Faculty of Medicine, Universitas Indonesia 95338, Jakarta, Indonesia; 5 Mahidol Oxford Tropical Medicine Research Unit 469893, Bangkok, Thailand; University of California San Francisco, San Francisco, California, USA

**Keywords:** malaria, *Plasmodium vivax*, latency, primaquine, 5,6-orthoquinone primaquine, CYP2D6, phenotype

## Abstract

Cytochrome P450 isotype 2D6 (CYP2D6) enzyme metabolizes the 8-aminoquinoline primaquine into its active form, 5-hydroxyprimaquine. Highly polymorphic CYP2D6 produces different enzyme activity levels among individuals. This diversity influences the therapeutic efficacy of primaquine in preventing *Plasmodium vivax* malaria relapses. Urinary 5,6-orthoquinone primaquine concentrations in Indonesian patients treated with primaquine for malaria were analyzed in relation to CYP2D6 genotype-predicted phenotypes and blood methemoglobin levels. We genotyped samples from 48 patients and classified them into three CYP2D6 metabolic activity groups: intermediate, normal, and ultra-rapid metabolizers. Methemoglobin levels were measured repeatedly up to 27 times over 72 h, including one pre-dose measurement and eight post-dose measurements per day at approximately 30-min intervals following primaquine administration. Intermediate metabolizers exhibited significantly lower urinary concentrations of 5,6-orthoquinone primaquine compared to normal and ultra-rapid metabolizers (geometric mean ratio: 2.24, 95% CI: 1.26–3.98, *P* = 0.01), indicative of reduced drug bioactivation. Methemoglobin concentrations gradually increased from Day 0 to Day 2, particularly in normal metabolizers. Day 2 methemoglobin levels were higher in normal/ultra-rapid metabolizers in our study sample (geometric mean ratio = 1.51; 95% CI: 0.84–2.70; *P* = 0.15). There was a strong positive relationship between urinary 5,6-orthoquinone primaquine and Day 2 methemoglobin levels (geometric mean ratio per 1,000 ng/mL increase = 1.90; 95% CI: 1.37–2.63; *P* = 0.0007). Our findings accord with the hypothesized utility of urinary 5,6-orthoquinone primaquine concentrations and methemoglobin levels as pharmacodynamic markers of primaquine bioactivation. Understanding and measuring the impacts of CYP2D6 polymorphisms will aid in interpreting clinical trials and optimizing primaquine dosing strategies.

**CLINICAL TRIALS:**

This study is registered with ClinicalTrials.gov as NCT03916003.

## INTRODUCTION

Primaquine (PQ) is an 8-aminoquinoline drug that has been used for over 70 years to treat the latent hepatic stage of *Plasmodium vivax* and *Plasmodium ovale* malaria (1). The activation of dormant hepatic forms of *P. vivax* and *P. ovale*, known as hypnozoites, leads to renewed malaria attacks commonly referred to as relapses. This is particularly critical in *P. vivax* malaria-endemic settings, where these hypnozoite-derived relapses, rather than new mosquito-borne infections (i.e., reinfections), account for over 80% of acute *P. vivax* malaria episodes (2). In a large cohort of Indonesian patients followed for 12 months, patients initially diagnosed with *P. vivax* malaria experienced significantly higher rates of hospital admission and all-cause mortality compared with patients diagnosed with *P. falciparum* malaria (3). Effective PQ treatment against relapses is thus a critically important clinical and public health objective. This therapy is often challenging and can be dangerous without screening for glucose-6-phosphate dehydrogenase (G6PD) deficiency—a common inherited blood abnormality that causes red blood cells to lyse when exposed to PQ, among other agents and conditions (4). At therapeutic dosing of 0.25 or 0.50 mg/kg/day, patients with G6PD activities less than 30% may develop acute hemolytic anemia, which may prove fatal (5).

Primaquine is a prodrug with a complex metabolic profile that yields many known metabolites. Its 5-hydroxylation by cytochrome P450 isotype 2D6 (CYP2D6) generates therapeutically active 5-hydroxyprimaquine (6, 7). The gene encoding the CYP2D6 enzyme is naturally polymorphic, with over 100 known alleles showing activity phenotypes that range from null to ultra-rapid metabolizers (8–10). The increasing CYP2D6 activity phenotypes positively correlate with the therapeutic success of PQ in preventing relapses (11, 12). The active 5-hydroxyprimaquine metabolite exists in redox equilibrium with its quinonimine oxidized derivative and is highly unstable; however, its degradation product, 5,6-orthoquinone primaquine (5,6-OQPQ), is stable and detectable in urine (13, 14). In Cambodian adults, urinary concentration of 5,6-OQPQ was significantly influenced by CYP2D6 metabolizer phenotype; normal metabolizers (NMs) exhibited nearly three times higher urinary 5,6-OQPQ concentration compared to intermediate metabolizers (IMs) (15). The prevalence of the CYP2D6*10 impaired-function allele was 57% among Cambodians, suggesting an elevated risk of PQ treatment failures. Urinary 5,6-OQPQ may serve as a biomarker for the extent of PQ metabolism and its treatment efficacy.

Methemoglobin levels in blood have emerged as a potential predictor of anti-relapse efficacy (16). While methemoglobin itself may not directly contribute to the elimination of hepatic hypnozoites, it may nonetheless reflect the presence and activity of the PQ metabolites involved in that elimination process (17). In a recent multi-country individual patient data meta-analysis (18), high methemoglobin 7 days after PQ dosing was associated with a reduced risk of *P. vivax* recurrence. Methemoglobin elevation may serve as a valuable proxy of likely PQ anti-hypnozoite activity.

In the current study, we examined the association between the CYP2D6 genotype-predicted phenotype and urine 5,6-OQPQ concentration in patients recruited into a malaria clinical trial in endemic settings and treated with PQ (19). In addition, we investigated the association between methemoglobin and 5,6-OQPQ in urine.

## RESULTS

A total of 48 patients were included in the analysis. Table 1 presents their demographic data stratified by sex. All patients were confirmed as G6PD-normal prior to their inclusion in this study. The participants were residents of the island of Sumba in eastern Indonesia. Ethnicity data were missing for 17 individuals, possibly because they were unsure of their ethnic origin, were migrants, or of mixed heritage from outside the island.

**TABLE 1:** Demographic data of patients with *Plasmodium falciparum^a^*

Characteristic	Sex	
	Male	Female
Number of individuals	21	27
Median age (years, min–max)	20.3 (3.5–79.4)	14.3 (1.7–53.8)
Median body weight (kg, min–max)	45.3 (14.0–65.4)	36.5 (10.7–54.0)
Median G6PD (U/gHb, min–max)	9.5 (6.0–13.8)	7.5 (5.9–12.1)
Ethnicity		
Sumba/Sumba Timur	16 (76.2%)	15 (55.6%)
Unknown	5 (23.8%)	12 (44.4%)

^*a*^ kg, kilogram; g, gram; U/gHb, unit per gram of hemoglobin.

### CYP2D6 genotype-predicted phenotypes

All 48 patients were successfully genotyped. Base substitutions, including indels, were determined at 11 regions (exons 1, 2, 3, 4, 6, 7, and 9; introns 1, 3, and 6) of the *CYP2D6* gene. Two of the included patients were related: a female patient with *1/*2 xN ultra-rapid metabolizers (UMs) was recorded as the mother of another female patient with *1/*10 NM genotype. To avoid overestimating allele frequency, the two shared alleles (*1) were combined into a single copy, resulting in a total of 95 alleles among the 48 patients.

We identified five CYP2D6 alleles: *1, *2, *10, *14, and *41. Additionally, we detected CYP2D6 *5 gene deletions and increased copy numbers (CYP2D6 *1 xN, *2 xN, and *10 xN). The decreased-function allele *10 with increased copy number was the most frequent (27.4%), followed by the wild-type allele *1 (21.1%) and the deletion of allele *5 (8.4%).

Table 2 lists the 15 distinct CYP2D6 genotypes observed. The most common genotype was *1/*10 xN, found in 27.1% of patients, followed by *1/*10 (16.7%) and *10/*10 xN (12.5%). These were as follows: 60.4% were NMs, 31.3% were intermediate metabolizers (IMs), and 8.3% were UMs. Eight IM patients were heterozygous for the CYP2D6 *5 allele, and seven other IM phenotypes were homozygous for the CYP2D6 *10 allele. Four patients carrying normal-function alleles (*1 and *2) with increased copy numbers were classified as UMs. None of the patients were classified as having a null metabolizer phenotype.

**TABLE 2:** CYP2D6 genotype frequencies and predicted phenotypes in 48 patients tested for 5,6-orthoquinone primaquine^*a*^

Genotypes	Number of patients	Genotype frequency (%)	Predicted phenotype	Range activity score (AS)	Lower estimate activity score (AS1)	Higher estimate activity score (AS2)
*5/*10	4	8.3	IM	0.25	0.25	0.25
*5/*41	1	2.1	IM	0.25	0.25	0.25
*10/*10	1	2.1	IM	0.5	0.5	0.5
*10/*10 xN	6	12.5	IM	0.75	0.75	0.75
*1/*5	3	6.3	IM	1	1	1
*1/*10	8	16.7	NM	1.25	1.25	1.25
*1/*10 xN	13	27.1	NM	1.5–2.25	1.5	2.25
*2/*10 xN	1	2.1	NM	1.5–2.25	1.5	2.25
*1/*14	1	2.1	NM	1.5	1.5	1.5
*1/*1	4	8.3	NM	2	2	2
*1/*2	1	2.1	NM	2	2	2
*2/*2	1	2.1	NM	2	2	2
*2/*2 xN	1	2.1	UM	3	3	3
*1/*1 xN	2	4.2	UM	3	3	3
*1/*2 xN	1	2.1	UM	3	3	3

^*a*^ IM, intermediate metabolizer; NM, normal metabolizer; UM, ultra-rapid metabolizer. xN refers to an increased copy number, which indicates that there are more than two copies, which could be due to a duplication of the CYP2D6 region. Estimation of activity scores is based on CYP2D6 genotypes. The lower estimate activity score is provided in cases where allele duplication (copy number variation/CNV) occurs, but the method used cannot determine which allele is duplicated. Therefore, two possible estimates are given: (i) Lower Estimate (AS1), if the allele with lower activity is duplicated, and (ii) Higher Estimate (AS2), if the allele with higher activity is duplicated. If the genotype is homozygous (e.g., *10/*10), the lower and higher estimates are the same.

The most common genotype was *1/*10 xN, observed in 13 patients (27.08%), with a predicted NM status and an activity score range of 1.5–2.25. One patient with *2/*10 xN also had an activity score range of 1.5–2.25 (Table 2). Other frequently observed genotypes included *1/*10, *1/*10 xN, and *1/*1, all predicted NM phenotypes. IMs were present in patients with genotypes *5/*10, *5/*41, *10/*10, *10/*10 xN, and *1/*5. UMs were less common but were observed in genotypes such as *1/*1 xN, *2/*2 xN, and *1/*2 xN.

#### Urinary 5,6-orthoquinone primaquine

**Fig 1:**
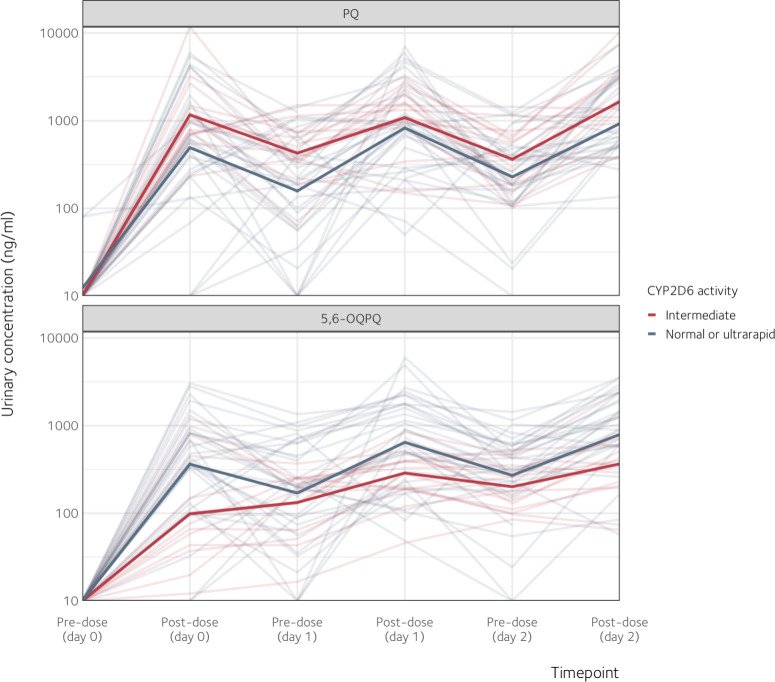
Temporal dynamics of urinary drug concentration and metabolite levels. Both analyte primaquine (PQ) and 5,6-orthoquinone (5,6-OQPQ) were measured on three consecutive days (day of enrollment, timepoints D0–D2) with pre- and post-dose concentrations per day. Each line represents individuals stratified by genotype-predicted phenotype: red (IMs) and blue (NMs/UMs). The solid line represents the average of concentrations of PQ and 5,6-OQPQ. The time interval between pre- and post-dose (3–6 h), and the median time interval between post- and pre-dose tis 17 (10–40) h. The *X*-axis is the time point of the day and dosage; the *Y*-axis is an absolute urinary concentration on a logarithmic scale.

An absolute urinary concentration of both PQ and 5,6-OQPQ temporal dynamic showed that four individuals’ urinary metabolite concentrations returned to baseline levels on the following day prior to drug administration; three of these individuals were NMs and one was a UM (Fig. 1). Subsequently, we observed an increasing trend in concentration for both analytes over a 3-day regimen, suggesting drug accumulation. Consistently, NM/UM subjects exhibit lower PQ concentration but higher 5,6-OQPQ concentrations compared to IMs. While no significant correlation was found between baseline PQ and daily peak (post-dose) PQ concentration (*r* = 0.193; 95% CI: 0.024–0.352; *P* = 0.026), a significant relationship was observed between pre- and post-dose concentrations of 5,6-OQPQ (*r* = 0.439; 95% CI: 0.291–0.567; *P* = 0.001) (Fig. S9). This suggests that an accumulation effect is more prominent in the metabolite’s dynamics than in the parent drug (Fig. 1).

Table 3 lists the urinary PQ and 5,6-OQPQ concentration in patients according to genotype-predicted phenotype. For statistical comparisons, independent samples *t*-tests were performed on log-transformed data on Day 1, comparing IMs vs NMs and UMs. For PQ concentrations in urine, there was no statistically significant difference between IMs and NMs/UMs. The geometric mean PQ concentration in IMs was 1,086.9 ng/mL (*n* = 15), while in NMs/UMs it was 882.4 ng/mL (*n* = 33). The geometric mean ratio (IMs vs NMs/UMs) was 1.23 (95% CI: 0.639–2.375; *P* = 0.52).

**TABLE 3:** Urinary primaquine metabolite concentrations and metabolic ratio data stratified by genotype-predicted phenotype^*a*^

	Genotype-predicted phenotype		
	IM	NM	UM
Number of patients	15	29	4
Male/female ratio	5/10	15/14	1/3
Median PQ dose (mg/kg)	1.02 (0.40–1.20)	1.07 (0.37–1.29)	1.05 (0.39–1.20)
Median PQ (ng/mL urine)	1493 (148–3,265)	897 (50–5,911)	865 (261–7,051)
Mean PQ (ng/mL urine)	1,443	1,624	2,261
Median 5,6-OQPQ (ng/mL urine)	327 (46–725)	895 (48–4,859)	1,202 (109–5,998)
Mean 5,6-OQPQ (ng/mL urine)	351	1,064	2,128
MR (PQ/5,6-OQPQ)	4.41 (0.42–14.34)	1.74 (0.12–4.81)	1.36 (0.46–6.48)

^*a*^ 5,6-OQPQ, 5,6-orthoquinone primaquine; IM, intermediate metabolizer; MR, metabolic ratio; NM, normal metabolizer; PQ, primaquine; UM, ultra-rapid metabolizer.

**Fig 2:**
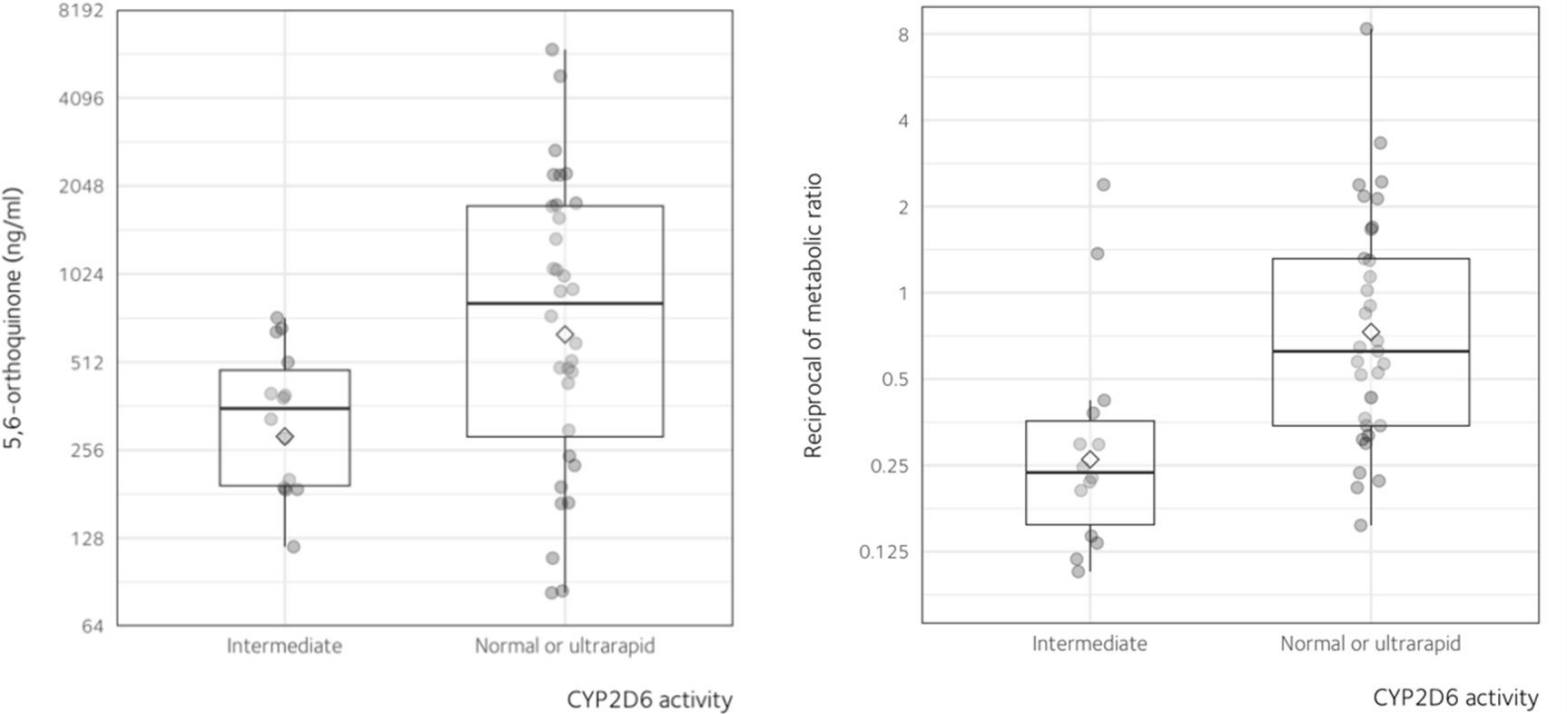
Boxplots of 5,6-OQPQ concentrations (left) and the reciprocal of metabolic ratio (5,6-OQPQ/PQ) (right) by CYP2D6 phenotype. 5,6-OQPQ concentrations vs CYP2D6 activity group: geometric-mean ratio 2.24 (95% CI: 1.26–3.98), *P* value 0.01, coefficient correlation: *r* = 0.35 (95% CI: 0.2–0.57), *R*^2^ = 0.11 (95% CI: 0.01–0.29). 5,6-OQPQ/PQ vs CYP2D6 activity group, geometric-mean ratio: 2.83 (95% CI: 1.54–5.04), *P* value 0.001, coefficient correlation: *r* = 0.50 (95% CI: 0.22–0.71), *R*^2^ = 0.11 (95% CI: 0.01–0.30).

Figure 2 illustrates the associations between 5,6-OQPQ concentrations measured in the urine of patients on Day 1. Classified as IMs vs NMs and UMs (left panel), along with the reciprocal of the metabolic ratios of the same patients, where lower values indicate low rates of metabolic conversion of 5,6-OQPQ to PQ (right panel). IMs showed significantly lower concentrations of 5,6-OQPQ in urine compared to NMs or UMs, 2.24 (95% CI: 1.26–3.98; *P* = 0.01). Whereas the reciprocal metabolic ratio of 5,6-OQPQ to PQ in urine was 2.83 (95% CI: 1.54–5.04, *P* = 0.001).

### Blood methemoglobin levels

**Fig 3:**
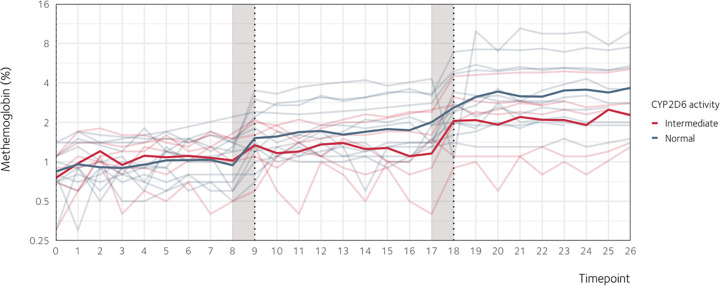
Temporal dynamics of blood methemoglobin levels. Methemoglobin levels were measured on Day 0 (day of enrollment, time points 0–8), Day 1 (time points 9–17), and Day 2 (time points 18–26); stratified by genotype-predicted CYP2D6 phenotype. Each transparent red or blue line corresponds to an individual. Solid red and blue lines correspond to the average at each time point. The time interval between consecutive timepoints is approximately 30 min, except for those shaded in gray, which represent several hours leading to the next day’s measurements (intervals between 8–9 and 17–18). Time points 0, 9, and 18 (dotted vertical lines) correspond to the daily pre-dose measurements. The vertical axis is shown on a logarithmic scale.

Repeated methemoglobin measurements were available for 18 patients throughout the 72-h duration of their admission to the clinical wards. We observed consistent increases in methemoglobin levels from Day 0 to Day 2 (Fig. 3; Fig. S1 to S3). However, within each day, this increase only became apparent after 4 h. Marked increases only became evident on the following day, including immediately before PQ was re-dosed. As the first 3 days passed since enrollment, a higher body weight-adjusted daily dose of PQ appears to result in an increased methemoglobin level, particularly among normal metabolizers (Fig. S4).

IMs appear to show lower methemoglobin responses to PQ compared to NMs and UMs, particularly on Day 2 (Fig. 3). However, we found little to no evidence of association between genotype-predicted phenotype and Day two methemoglobin levels (geometric mean ratio for NMs vs. IMs = 1.51 (0.84, 2.70); *P* = 0.15; correlation coefficient = 0.36 (–0.11 to 0.72), *R*^2^ = 0.12 (0.00–0.44)). On Days 0 and 1, this pattern was less apparent (Fig. S5). Similar to methemoglobin, a higher body weight-adjusted daily dose of PQ appears to result in an increased 5,6-OQPQ concentration, particularly among NMs or UMs (Fig. S6).

We observed a positive relationship between 5,6-OQPQ and Day 2 methemoglobin levels. For each additional 1,000 ng/mL increase in 5,6-OQPQ concentration, we found an approximately 90% higher Day 2 methemoglobin level (geometric mean ratio per 1,000 ng/mL increase = 1.90 [1.37–2.63]; *P* = 0.0007; correlation coefficient = 0.72 [0.37–0.90], *R*^2^ = 0.52 [0.14–0.80]). Figure S7 shows the model implied prediction across 5,6-OQPQ concentrations (refer to Fig. S8 for the fitted line displayed on a logarithmic scale, which highlights the linear trend).

## DISCUSSION

We demonstrate a correlation between CYP2D6 genotype-predicted metabolizer phenotypes and 5,6-OQPQ concentration in urine for three consecutive days of daily PQ dosing. Patients predicted by genotypes to have intermediate metabolism of PQ to 5,6-OQPQ (IMs) showed that they had consistently lower urinary 5,6-OQPQ concentration compared to concentrations in NMs/UMs volunteers (Fig. 2). We favored the reciprocal of the metabolic ratio of PQ/5,6-OQPQ (i.e., ratio of 5,6-OQPQ to PQ) in urine as an indicator of relative metabolic activity, where low vs high values reflect intermediate metabolic activity. At yet undefined thresholds of that inverse ratio, patients may be at relatively high risk of therapeutic failure of PQ against latent malaria. Larger studies of PQ/5,6-OQPQ concentration in urine linked to such therapeutic outcomes may be required to establish these thresholds, which were outside the scope of this work. Still, our findings nonetheless affirm the relationship between naturally variable CYP2D6 activity and the generation of the therapeutically active species of PQ; the unstable 5-hydroxyprimaquine metabolite degraded to stable 5,6-OQPQ (7, 20).

In a previous individual patient data meta-analysis, most patients receiving daily PQ dosing for latent malaria experience a gradual rise in methemoglobin levels that reach plateau levels after about 7 days. Day 7 methemoglobin levels were shown to be associated with a reduction in vivax recurrence (17). In this study, blood methemoglobin accumulation was successfully monitored during the first 72 h of PQ therapy in 18 of the recruited patients. We documented the stability of the methemoglobin signal within individual patients and through three daily dosing cycles. Higher levels of blood methemoglobin appeared to be associated with both the CYP2D6 genotype-predicted phenotype and that of increased concentrations of 5,6-OQPQ in their urine. These preliminary findings align with post-PQ dosing methemoglobin levels, indicating the likelihood of therapeutic success or failure, possibly as early as 72 h after commencing treatment. Our hypothesis requires validation in larger trials involving actual vivax malaria patients. By monitoring blood methemoglobin and corrected urinary creatinine 5,6-OQPQ monitoring up to day seven, this would allow us to observe the dynamic formation of methemoglobin and 5,6-OQPQ more clearly. Given that methemoglobin peaks at Day 7 and 5,6-OQPQ accumulates over time, correlating these markers with patient outcomes is the next step to confirm our findings.

The ability to effectively treat latent vivax malaria impacts the ability to control and possibly eliminate malaria from any given endemic zone. However, it is also a vitally important clinical objective for individual patients. Repeated malaria attacks at relatively brief intervals deepen illness and the gravity of consequences to patients (3). Most of the global burden of *P. vivax* malaria occurs in South and Southeast Asia (21–23), where the frequency of the intermediate CYP2D6 allele *10 exceeds 50% (24, 25). Our limited survey of CYP2D6 genotypes of people living on Sumba is in accordance with those broader and more extensive surveys; 33 of the 48 patients (69%, 95% CI: 55–80%) carried the *10 allele. In all, 15 of our patients (31%) had CYP2D6 genotypes consistent with the IMs, and most of them had low concentrations of PQ metabolism to 5,6-OQPQ.

Our study has several limitations. Firstly, the sample size was limited to forty-eight patients with acute *P. falciparum* malaria (19). We could only examine the excretion of PQ and 5,6-OQPQ, along with methemoglobin measurements in 18 patients, during the first 3 cycles of daily dosing. Second, the patients recruited in this study were positive for *P. falciparum* rather than *P. vivax*. No patients experienced post-treatment *P. vivax* episodes within the observation period of 63 days, so we cannot directly link our findings to actual therapeutic outcomes (26). The poor metabolizer phenotype was apparently too rare for capture in our sample. Third, our methemoglobin measurements were limited to the first three days of daily dosing. The lack of measurements on Day 7, which is generally considered the optimal time to correlate pharmacokinetic (PK) parameters such as AUC (Area Under the Curve) and drug exposure, with therapeutic response. Yet, with the limited sample size, our study offers a quantitative PK/PD interpretation, by directly associating CYP2D6 genotype, metabolite exposure, and methemoglobinemia.

The availability of a validated metabolic biomarker predictive of hypnozoite clearance after initiating PQ treatment would provide a biosurveillance tool to assist national malaria control programs in eliminating relapsing vivax malaria. Our findings point to two possible indicators: (i) high concentrations of urinary 5,6-OQPQ relative to PQ in urine and (ii) high levels of blood methemoglobin. These two markers represent the initial step towards developing a point-of-care rapid diagnostic test (RDT) aimed at identifying individuals who are at a higher risk of therapeutic failure or malaria relapse due to lower metabolic conversion to 5,6-OQPQ. If this quantitative validation can be performed more broadly, in terms of both sample size and population diversity, we can develop tools such as a urine dipstick or other RDTs. This tool would be based on the cut-off values of 5,6-OQPQ or methemoglobin concentration following PQ administration, an approach that is far more accessible for use in remote, malaria-endemic areas compared to genotype analysis or metabolite assays. By utilizing these markers, future trials can better account for inter-individual variability and establish therapeutic thresholds linked to patient outcomes.

## MATERIALS AND METHODS

### Study profile and participant selection

The current work was conducted in patients recruited at the Indonesian site into the PRIMA clinical trial (NCT03916003, INA-BB6K0Q7), which aimed to assess the efficacy and safety of high-dose PQ to reduce the risk of subsequent vivax malaria in patients presenting with falciparum malaria. Relevant ethics approvals include the Human Research Ethics Committee of the Northern Territory Department of Health (Menzies School of Health Research; reference number: 2019-3288), the Research Ethics Committee of the Faculty of Medicine, Universitas Indonesia (reference number: 20-03-0370, 2020), and the Oxford Tropical Research Ethics Committee (OXTREC) (reference number: 65-19). The funding for the PRIMA trial was provided by the Australian Academy of Science Regional Collaboration Program, Bill & Melinda Gates Foundation (INV-010504), and the Australian National Health and Medical Research Council (APP1132975). This study was supported by Wellcome Trust Grant (B9R05430).

Patients visiting the primary health clinics (i.e., PRIMA Indonesia study sites) in East Sumba, East Nusa Tenggara Province, Indonesia, were screened for uncomplicated *P. falciparum* malaria (19). Forty-eight eligible individuals diagnosed with uncomplicated *P. falciparum* malaria were recruited into the intervention arm of the study, which received dihydroartemisinin-piperaquine (DHA-PP) and high-dose PQ (1 mg/kg) administered once daily for 7 days. Study participants were admitted to the clinic for continuous observation for 3 days after initiation of treatment. The detailed recruitment process for the PRIMA trial, including informed consent and providing blood and urinary samples, has been described previously.

### Urine and blood collection

We collected urine samples immediately before and 3–6 h after each dose of PQ (15). At each time point, approximately 50 mL of urine was collected. Over the 3-day period of inpatient observation, each patient contributed six urine samples (one pre- and one post-dosing sample per day). After collection, urine samples were promptly stored on-site at –20°C. The samples were transported to Jakarta, where the central laboratory for sample processing is located, under temperature-controlled conditions and stored at –80°C until analysis. A total of 0.75 mL of venous blood was collected using an EDTA tube. Following collection, the sample was processed to obtain plasma and buffy coat and was initially stored at −20°C as a temporary storage before being sent to Jakarta for long-term storage at −80°C until analysis.

### Urinary 5,6-orthoquinone primaquine

#### Materials and reagents

Primaquine diphosphate (C_15_H_21_N_3_O·2H_3_PO_4_; MW = 455; primaquine MW = 259) was obtained from Sigma-Aldrich (St. Louis, MO, USA). 5,6-orthoquinone primaquine (C_14_H_17_N_3_O_2_; MW = 259.31) was provided by Walter Reed Army Institute of Research (WRAIR). Stable isotope internal standards for primaquine (PQ-d3) and 5,6-orthoquinone primaquine (5,6-OQPQ-d6) were purchased from Toronto Research Chemicals (Canada). MS grade methanol, acetonitrile, water, and formic acid were from Sigma-Aldrich.

#### Chromatography and mass spectrometry

The analysis of PQ and 5,6-OQPQ was performed using a liquid chromatography-tandem mass spectrometry (LC-MS/MS) system (Agilent Infinity II 1290). Chromatographic separation was performed using Synergy C18 (50 × 2.1 mm, 1.8 µm, 100 Å) from Phenomenex. The column temperature was maintained at 37°C with a gradient mode of mobile phase A (0.1% formic acid in water) and mobile phase B (0.1% formic acid in methanol) at a flow rate of 0.2 mL/min. The injection volume was 2 µL. The solvent for syringe washing was 100% methanol. The column was equilibrated for 10 min. Mass spectrometry was performed using AB Sciex TQ4500 with an electrospray ionization system at 4,000 V in positive ion mode. The curtain plate nitrogen stream was set to 35 units, and the vaporizer temperature was set at 400°C. Gas streams 1 and 2 (GS1 and GS2) were set to 50°C and 60°C, respectively. Collision gas was used at 1.8 mTor. The collision energy was set to 30 eV. The mass transitions are PQ (*m/z* = 260.1 > 147.2), for primaquine internal standard PQd3 (*m/z* = 263.2 > 190.2), and 5,6-OQPQ (*m/z* = 260.1 > 147.2), for orthoquinone primaquine internal standard 5,6-OQPQ-d6 (*m/z* = 266.1 > 147.1). Analyst Software (version 1.63) was used for tuning parameters, and Multiquant (version 3.0.2) was used for quantification.

#### Method validation

The validation method followed FDA guidelines (27, 28). The validation parameters included accuracy, precision, linearity, selectivity, specificity, stability in the autosampler chamber, matrix effects, and recovery. Both lower limit of quantification (LLOQ) and upper limit of quantification (ULOQ) for PQ and 5,6-OQPQ are 10 and 1,000 ng/mL. Intra-day and inter-day assay precision and accuracy, expressed as the %CV of quality control samples at low (20 ng/mL for both PQ and 5,6-OQPQ), middle (125 ng/mL for both PQ and 5,6-OQPQ), and high (700 ng/mL for both PQ and 5,6-OQPQ) concentrations, were <15%. All standard and internal standard stock solutions were made separately in HPLC-grade pure methanol and maintained at −80°C, covered with aluminum foil to protect against light. Selectivity and specificity were assessed by examining the replicated response in the blank urine matrix and blank urine spiked with the internal standard. The results should show no interference at the retention times of the analytes (PQ and 5,6-OQPQ).

#### Sample preparation

Urine (100 µL) was spiked with 20 µL of deuterated internal standard (IS) PQ-d3 and 5,6-OQPQ-d6 (5 ppm each), briefly mixed, and spun down. Added 460 µL cold acetonitrile, mixed, and centrifuged at 12,000 × *g* for 5 min. The samples were added with 200 µL of methanol, mixed, and centrifuged at 12,000 × *g* for 5 minutes. The upper 650 µL layer was collected and transferred into an amber HPLC vial before being injected into the liquid chromatography system. All sample preparation was conducted under cold conditions (2–10°C) (14, 15). A sample analysis was performed using Multiquant Software.

### CYP2D6

#### DNA extraction

DNA was extracted using QIAamp DNA Mini Kit (Qiagen cat #51306) from 100 µL of buffy coat collected on the day of enrollment (Day 0). DNA was eluted in 100 µL of nuclease-free water, then measured using a spectrophotometric method (Implen NanoPhotometer N80) to determine its concentration and purity. The ratio of ultraviolet (UV) spectrophotometric readings at 260 and 280 nm wavelengths was between 1.7 and 2.0 (i.e., *A*_260_/*A*_280_ = 1.7–2.0).

#### CYP2D6 SNP genotyping and CNV assays

Amplification, purification, and single-base nucleotide extension were performed using Veridose Core Panel (Agena Bioscience cat #13250). Assays were performed following the manufacturer’s instructions (29, 30). It directly interrogates 18 single-nucleotide polymorphisms (SNPs) and indels, as well as 5 copy number variants (CNV), specifically targeting CYP2D6—the gene of primary interest in this study; the genetic markers correspond to 22 variants alleles (*1, *2, *3, *4, *5, *6, *7, *8, *9, *10, *11, *12, *14, *15, *17, *18, *19, *20, *29, *41, *69, and *114). CNVs were detected using the 2N control Veridose CYP2D6 CNV Panel (Agena Bioscience cat #2005). Most individuals carry two copies of each gene, but events such as gene deletion or duplication can result in 0, 1, or 3+ copies per gene in the genome. Sample dispensing was performed using Agena Nanodispenser RS1000, and genotyping was conducted using Agena MassARRAY Analyzer (San Diego, CA, USA). Predicted phenotypes were determined using the activity score (AS-A). AS-A estimates relative enzyme capacity based on genetic variations, assigning numerical values (from 0 to 2 or higher) to CYP2D6 allelic combinations, giving an objective means of classifying the predicted CYP2D6 phenotype.

#### Data analysis

Genotype variants were analyzed using Typer Analyzer version 4.1 (Agena Bioscience). Phenotype translation was performed using the activity score model described by the Clinical Pharmacogenetics Implementation Consortium (CPIC) and PharmGKB, with the following allele scores: 0.0 for *3, *4, *5, *6, *7, *8, *11, *12, *15, *18, *19, *20, *69, and *114 alleles; 0.25 for the *9, *10, and *41 alleles; 0.5 for *14, *17, and *29 alleles; and 1.0 for *1 and *2 alleles. The sum of the two alleles determined the predicted activity score phenotype of the individual. UMs have a diplotype with an activity score greater than 2.25. NMs have an activity score of 1.25–2.25, while IMs have an activity score of 0.25–1. Individuals with poor metabolizer (PM) status have an activity score of 0 (8, 20).

### Blood methemoglobin

Methemoglobin measurement was performed directly at the bedside over the first three days using the Masimo Rad-57 Pulse CO-Oximeter. The measurements were taken prior to the first PQ administration on each day (pre-dose) and subsequently up to 4 h following PQ dosing with 30-min intervals (post-dose), resulting in 27 measurements over 72 h. The first measurement served as the baseline value to assess changes in methemoglobin levels throughout the 4-h period on each day and the 72-h period.

### Statistical analysis

The statistical regression analyses examine the correlation between CYP2D6 genotype/phenotype status with urinary 5,6-OQPQ concentration and compare this with the reciprocal metabolite ratio (ratio of 5,6-OQPQ to PQ). Reciprocal metabolic ratio was used to facilitate the statistical model by changing the naturally negative correlation to a positive correlation for more intuitive interpretation of the rate of bioactivation. Additionally, we assessed the association between CYP2D6 genotype/phenotype status and methemoglobin levels. We also investigated the association between urinary 5,6-OQPQ and Day 2 blood methemoglobin concentrations. Day 1 PQ and 5,6-OQPQ concentrations were specifically chosen as they represented the most complete data set (46/48 = 95.8%) and demonstrated optimal distribution characteristics. Missing data on Day 1 were imputed using the average measurement from Day 0 and Day 2. All association analyses between absolute concentration 5,6-OQPQ and genotype-predicted phenotype were based on these selected Day 1 PQ and 5,6-OQPQ concentrations. Methemoglobin measurements were available from 18 patients (18/48 = 37.5%). One patient on Day 0 had a methemoglobin level of approximately 7% at a single time point, which was likely a misreading of the device; therefore, this value was excluded from further analysis. Changes in blood methemoglobin levels are reported over time after primaquine dosing between Days 0 and 2, separately for the CYP2D6 activity groups (intermediate and normal). Descriptive plots in association with PQ dose were also explored.

During a 7-day or 14-day primaquine regimen administered daily, methemoglobin levels are known to accumulate and peak around 1 week (18, 31, 32). In our sample, greater individual variability and the highest increases in methemoglobin levels were observed by Day 2. Therefore, we decided to use the maximum methemoglobin level on Day 2 as our summary metric in our models. We compared Day 2 methemoglobin levels between individuals with IMs and NMs CYP2D6 phenotypes to evaluate the association between these two biomarkers. By applying a logarithmic transformation to methemoglobin levels, we estimated the geometric mean ratio using the *t*-test with equal variances. We also estimated the association between Day 2 methemoglobin levels and 5,6-OQPQ concentration by regressing Day 2 methemoglobin levels on 5,6-OQPQ using log-linear regression

Statistical analysis, along with data visualization, was performed using R Statistical Software (version 4.3.0). The code for data analysis and visualization can be found at https://github.com/ihsanfadil/methb-orthoq.

## SUPPLEMENTAL MATERIAL

Fig. S1 aac.00539-26-s0001.tiff Temporal dynamics of methaemoglobin levels, summarised at each timepoint using various summary metrics.

Fig. S2 aac.00539-26-s0002.tiff Temporal dynamics of methaemoglobin levels, summarised at each timepoint.

Fig. S3 aac.00539-26-s0003.tiff Changes in methaemoglobin levels for each individual.

Fig. S4 aac.00539-26-s0004.tiff The body weight-adjusted daily dose of primaquine, stratified by the day of measurement and CYP2D6 phenotype.

Fig. S5 aac.00539-26-s0005.tiff Methaemoglobin and 5,6-orthoquinone concentrations, stratified by the day of measurement.

Fig. S6 aac.00539-26-s0006.tiff Body weight-adjusted daily dose of primaquine and urine 5,6-orthoquinone, stratified by CYP2D6 phenotype.

Fig. S7 aac.00539-26-s0007.tiff Association between urine 5,6-orthoquinone and day-2 blood methemoglobin concentrations.

Fig. S8 aac.00539-26-s0008.tiff Association between urine 5,6-orthoquinone and day-2 blood methaemoglobin concentrations.

Fig. S9 aac.00539-26-s0009.tiff Relationship of pre- and post dose.

ASM does not own the copyrights to Supplemental Material that may be linked to, or accessed through, an article. The authors have granted ASM a non-exclusive, world-wide license to publish the Supplemental Material files. Please contact the corresponding author directly for reuse.
